# How do I manage functional visual loss

**DOI:** 10.1038/s41433-024-03126-w

**Published:** 2024-05-22

**Authors:** Neil Ramsay, Justin McKee, Gillian Al-Ani, Jon Stone

**Affiliations:** 1 Institute of Neurological Sciences, Queen Elizabeth University Hospital, Glasgow, UK; 2 Dept Ophthalmology, Royal Infirmary of Edinburgh and Princess Alexandra Eye Pavilion, Edinburgh, UK; 3 Centre for Clinical Brain Sciences, University of Edinburgh, Edinburgh, UK

**Keywords:** Rehabilitation, Education

## Abstract

Functional visual loss is a subtype of functional neurological disorder (FND) and is a common cause of visual impairment seen in both general and neuro-ophthalmological practice. Ophthalmologists can generally diagnose functional visual loss reasonably confidently but often find it harder to know what to say to the patient, how to approach, or even whether to attempt, treatment. There is little evidence-based treatment despite studies showing up to 60% of adults having impactful symptoms on long-term follow-up. The last 20 years has seen large changes in how we understand, approach, and manage FND more widely. In this article, we set out our practical approach to managing functional visual loss which includes : 1) Make a positive diagnosis based on investigations that demonstrate normal vision in the presence of subjectively impaired vision, not just because tests or ocular exam is normal; 2) Explain and label the condition with an emphasis on these positive diagnostic features, not reassurance; 3) Consider eye or brain comorbidities such as migraine, idiopathic intracranial hypertension or amblyopia; 4) Consider working with an orthoptist using diagnostic tests in a positive way to highlight the possibility of better vision; 5) Develop simple treatment strategies for photophobia; 6) Consider psychological factors and comorbidity as part of assessment and therapy, but keep a broader view of aetiology and don’t use this to make a diagnosis; 7) Other treatment modalities including hypnotherapy, transcranial magnetic stimulation and more advanced forms of visual feedback are promising candidates for functional visual loss treatment in the future.

## Abstract (zh)

功能性视力损失是功能性神经障碍(FND)的一种亚型, 也是综合眼科和神经眼科常见的视力损害原因。眼科医生通常可以十分自信地诊断功能性视力损失, 但往往难以弄清如何向患者解释, 如何处理, 甚至是否尝试治疗。

尽管研究显示高达60%的成年人在长期随访中出现症状, 但几乎没有循证治疗。过去的20年里, 我们在更广泛地理解、处理和管理FND方面发生了巨大变化。本文阐述了功能性视觉损失的管理的实用方法, 包括: 1)在主观视力受损的情况下, 根据显示视力正常的检查做出阳性诊断, 而非仅因为眼科检查提示正常；2)强调用阳性诊断特征来向患者解释和标记病情, 而不是口头上的保证；3)需考虑眼或脑的合并症, 如偏头痛、特发性高颅压或弱视；4)需考虑与斜视医生的合作, 积极使用诊断测试, 以突出改善视力的可能性；5)针对畏光症制定简单的治疗措施；6)评估和治疗时应考虑心因性疾病, 但要从更广泛的角度看待病因, 不要依此诊断；7)其他治疗方式包括催眠疗法、经颅磁刺激和更高级的视觉反馈形式, 未来有望成为治疗功能性视力损失的备选方案。

## Introduction

Functional visual loss like other subtypes of functional neurological disorder (FND) involve the subjective experience of visual impairment in the presence of findings that demonstrate that visual pathways in the eye and brain are structurally intact, sometimes called internal inconsistency [1]. The brain of the person with functional visual loss won’t let them have the visual experience that they ought to be able to have.

In the ophthalmology literature, there is often an incorrect assumption that functional visual loss, in which the person experiences the symptoms, and simulated or malingered visual loss, where the person deliberately pretends to have symptoms, are strongly overlapping phenomena. Feigned, factitious or malingered factitious visual loss does also occur and is discussed below but is much rarer than functional visual loss.

A recent survey of 119 UK ophthalmologists found that 26% did not feel comfortable diagnosing functional visual loss, 59% felt they would not be confident in discussing functional visual loss and 58% felt they would struggle to discuss associated psychological factors if present [2]. It highlighted ambivalent attitudes to management with 37% stating that patients should not be able to claim disability benefits because it may prevent recovery. Many felt they lacked the space and time to provide treatment as well as concerns about accessing appropriate psychological input.

Most articles in this area have focused on diagnosis and the means to provide positive evidence of visual tract function despite symptomatic visual loss. Descriptions of treatment often involve simple reassurance from the ophthalmologist or referral to a psychiatrist.

This review aims for something different in providing a practical approach to the management of the patient once the diagnosis has been made. We have focused especially on management that ophthalmologists or neuro-ophthalmologists can get involved with, including using tests as part of the explanation and potential therapies to improve visual function. We have restricted ourselves to functional visual loss only and do not discuss functional disorders of eye movement, or visual hyperphenomena such as visual snow. We have provided two case vignettes (Box 1 and 2).

Box 1 Migraine and bilateral functional visual loss A 19-year-old female presented with progressive photophobia and severe migraine with visual aura [43]. After two weeks she experienced persistent blurred vision in her right eye followed by her left. She spent most of her time in her bedroom in the dark because of migraine. One day she woke and had no vision apart from a faint glow. Migraine improved. She was told that she had functional blindness because of the normal investigations and that it was a psychological problem but was not persuaded this was correct.

Six months later she was referred for another opinion. We were able to demonstrate normal optokinetic nystagmus and asked her mother to use this at home to show the rest of the family how her brain could be made to respond to visual stimuli. We formulated her headache as a consequence of migraine and photophobia, her brain had shut down visual input to try to protect her from headache. We encouraged the family to look for occasions when the patient could see things and feed this back to her. Three sessions of transcranial magnetic stimulation were received positively by the patient who was surprised at the visual experience of phosphenes that it gave her. The patient also found four sessions of hypnotherapy helpful and considered they had ‘stirred something’ in her, although there was no immediate improvement. About 1 month later she found that she started to see more light with patterns of spots and after another month woke up to discover she had normal vision. Complete recovery has persisted at 5 year follow up.

Box 2 Functional visual loss with other types of FND A 16-year-old male presented with a history of visual impairment since the age of 12. He had been diagnosed 6 months earlier with a post fracture complex regional pain syndrome affecting the left leg with redness, swelling, dystonia and weakness. He also had anxiety, PTSD and was being assessed for autism spectrum disorder. He was put on Pregabalin and experienced visual blurring and dizziness and visual blurring that was initially intermittent but gradually became permanent. He reported being able to see colours and shapes but not letters. Vision is worse for moving objects. MRI brain, ERG, OCT and VEPs were normal and visual acuity was demonstrated as transiently normal using a prism examination.

He didn’t need much persuading that the diagnosis was functional visual loss having read already about the connection between CRPS and FND but was desperate to have improved vision. We experimented with reading words on paper that he couldn’t see at all, and he had to guess what they were. ‘Dog’ was guessed as ‘Cat’ and ‘Round’ was guessed as ‘Circle’ and we were able to reflect on how that indicated that there was a route from her eye to her brain that he wasn’t aware of. We also discussed times when he was able to read signs and tended to get a headache and how those indicated that we were challenging the block in his brain. He reported feeling very positive with the consultation. He found a discussion about the film Inside Out especially helpful. He is currently having hypnotherapy as well as psychotherapy for PTSD and visual acuity has improved. We have had frank conversations about how he might explain recovery of visual function to friends and family who might be surprised that he had improved.

## How common is functional visual loss and when does it need treatment?

Functional visual loss, and especially functional visual *signs* are common. One of the best studies found that it accounted for 12% (*n* = 133) of 1108 new neuro-ophthalmology patients to a centre in Oregon [3] with 57% female and 14% younger than 18 years old. The same study found that 53% had co-existent eye or brain disease that led to comorbid neuro-ophthalmological findings. 14% had experienced head or eye trauma within the past month and 14% had experienced a recent surgical procedure.

A 2016 systematic review of the prognosis of functional visual loss included 5 studies (between 1966 and 1989) with 132 patients, mostly adults, with a follow up over several years. Between 46 and 78% of patients had improved or remitted at follow up [4]. A further study of 8 cases in children showed that 25% had recovered in 1 month [5].

Our own experience is that only a proportion of patients with functional visual signs actually want or need further treatment for associated difficulties with acuity or visual fields. A patient’s poor performance during a test may not correlate with their difficulties day to day, which may indeed be partly how the diagnosis is made. For example, a patient with idiopathic intracranial hypertension who has spiralled visual fields but doesn’t complain of much in the way of visual symptoms does not need a detailed explanation or management of functional visual loss.

## Understanding aetiology and mechanism to enable explanation of functional visual loss

A clinician cannot be expected to be able to manage or explain a condition if they don’t have some understanding of it themselves. For many ophthalmologists, functional disorders are a ‘black box’ that they haven’t had training in where simplistic ideas (i.e. that it’s a purely psychological problem) pervade. This can lead clinicians to feel that it’s ‘not my problem’, or conversely to try to approach the problem in a way that doesn’t capture what we know about the complexity of what causes these symptoms.

**Fig. 1:**
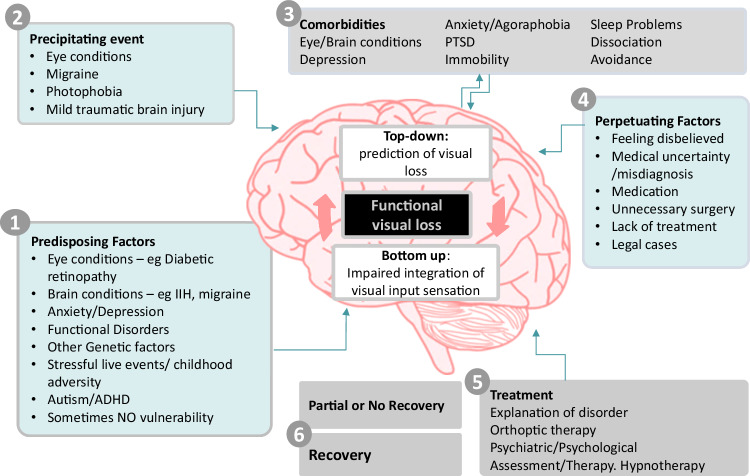
A predisposing, precipitating and perpetuating model of aetiology can combine with a mechanistic understanding of functional visual loss at the level of the brain.

Figure 1 indicates a more nuanced way to think about the aetiology and mechanism of functional visual loss. There are a range of possible aetiological risk factors which include eye and brain comorbidities as well as psychological risk factors such as adverse experience. Many of these can operate as predisposing, precipitating and perpetuating factors. Several studies have highlighted how commonly functional visual loss co-exists with neurological conditions such as migraine and idiopathic intracranial hypertension (IIH) and as well as other eye conditions (i.e. diabetic retinopathy or glaucoma). In one small series, 25% of patients had another neurological condition [6]. This comorbidity of other eye and brain conditions with functional symptoms and signs is often called ‘functional overlay’. We prefer the term ‘functional disorder comorbidity’, or dual diagnosis to emphasise the positive identification of a separate comorbidity with a different range of treatment. We don’t after all talk about ‘depression overlay’.

Studies in FND generally have indicated that there is a higher frequency of adverse experience, especially emotional neglect in people with the condition [7], although data in people with functional visual loss is sparse. One study of 36 patients found that only 20% had experienced a recent stressful event [6], although there are also examples of functional blindness occurring in clusters related to stress, for example in refugees from the Cambodian war [8]. Adverse childhood experience can lead to an amplified dissociative response to threat or may be associated with difficulty labelling emotions that could lead later to bodily sensations of distress not being recognized and being amplified. Recent research has highlighted autism spectrum disorder and attention deficit hyperactivity disorder (ADHD) as risk factors for FND. Autism is also associated with increased sensory sensitivity which may be especially important in promoting a ‘shut down’ response of the visual brain in response to threat.

There are only very limited studies of neural correlates in functional visual loss. A functional MRI study of five patients with functional visual loss and seven controls found reduced activation in visual cortices but increased activation in left frontal cortex, insula, bilateral striatum, left limbic structures and left posterior cingulate cortex [9]. Wider studies of people with motor FND are beginning to find convergent evidence of abnormalities in several brain networks, especially those related to the sense of agency (i.e., the parts of the brain that give you the sense that you are the agent of your own actions and sensations), attention (i.e., ability to respond to stimuli), and emotion) [10].

Another useful way to think about FND is a disorder of the predictive brain [11]. A wide range of evidence suggests that the brain works on a day-to-day basis by predicting action and sensation and then, after the event, checking whether sensory input matches the prediction. Most of the time it matches very well, and we have a seamless experience of agency. However, a range of disorders, especially in neurology and psychiatry make sense as disorder of this predictive processing. For example, in phantom limb phenomena, the brain predicts that a limb is still there, even though there is ‘bottom-up’ sensory input telling the brain otherwise. The prediction is so strong that it overrides sensory input. Similarly, in functional visual loss, it may be that the brain is predicting visual loss so strongly that it ignores visual input that should be updating that prediction.

Strong predictions are not necessarily ‘beliefs’ about blindness, they are neural models that may arise for maladaptive reasons, for example, to shut down visual input when it’s not wanted, for example because of photophobia.

Feigning remains a notable concern in ophthalmology, perhaps more so than in other disciplines. This may be because the tests so definitely prove that vision is present when the patient says it is not. Feigning and malingering does occur, especially in legal scenarios, but remains rare in clinical practice. A recent review article explained the numerous clinical and neuroscience reasons why feigning is such a poor explanation for the majority of FND symptoms that present in clinical practice [12]. James Paget’s 1873 quote about a female patient with ‘hysterical paralysis’; – ‘*She says I cannot, it looks like I will not, but it is I cannot will’* – has not been bettered. In other words, functional neurological symptoms may look wilfully exaggerated, but they are instead a disorder of higher volitional control and agency of movement and sensation.

## Diagnosis by inclusion – treats not tricks

The method of diagnosis depends on the severity of the visual impairment and whether monocular or binocular. Assessment of patients should be tailored to each situation. Assessment by an experienced orthoptist can be invaluable.

Examples of tests are shown in Table 1. Other reviews go through the details of these diagnostic tests in more detail, and many will be familiar to experienced ophthalmologists or neuro-ophthalmologists [13, 14].

**Table 1:** Positive diagnostic tests for functional visual loss and explanatory/therapeutic opportunities they provide.

Severe bilateral visual impairment	How to use the clinical feature therapeutically as part of explanation
Observation of the patient’s **ability to navigate** obstacles in the waiting room and clinic room	Consider video recording the patient’s gait to help persuade carers that vision may be better when navigating than at other times. This is good because it shows the potential for improvement
Observation of patient’s **ability to use a mobile phone** in waiting room and clinic including assessment of font size	Discuss how the font size they can see on their phone is better than their near vision achieved on formal testing. This shows that their brain can see better when they are relaxed and looking at interesting content compared to doing a stressful eye test
**Observing eye contact**. Often starts off with avoidance of eye contact initially which improves when more distracted or discussing things unrelated to visual loss.	Discuss with the patient and carer that you noticed this, and that good eye gaze depends on good vision. Discuss when eye gaze became better and how recreating this feeling may help in therapy.
**Optokinetic drum eliciting horizontal jerk nystagmus** (for those who have vision between no perception and hand movement) = acuity of at 6/120	Show carers how an optokinetic drum (or even an iPhone video of one) can induce nystagmus and thus provides evidence of a pathway between the eye and brain
**Mirror Gazing**. Ability to follow a reflection in a mirror that’s being rotated also denotes vision better than light perception	This may be more useful as a therapeutic manoeuvre similar to the optokinetic drum than a diagnostic one.
**Moderate bilateral visual impairment**	
**Modification of visual acuity checking** – for example start with smallest type and work upwards, or use single letter optotype, removing cue of other letters about what is ‘hard’	Consider doing the test in the standard way afterwards and share with the patient how they found it easier to see when not confronted by a task of increasing difficulty.
Lack of improvement in acuity with **moving the chart nearer**.	Explain how vision should improve in this situation but in functional visual loss, the brain “expects” to see badly regardless of how the image is present.
Use of **technology that promotes saccadic eye gaze** – eg ‘peekaboo vision’ app designed for young children that produces saccades towards a striated target at higher acuities than they are aware of, even if they can’t tap the correct target.	This can be explained similarly to nystagmus induced by an optokinetic drum (see above). Your brain IS seeing the target, enough to move your eyes towards it, but not enough to give you a signal to make a tap.
**Moderate or Severe Unilateral Visual Impairment**	
**Fogging test** in which vision in the good eye is worsened to show that vision must be coming from the bad eye by: a) progressively fogging the good eye b) blocking vision with an over-powered refractive correction (eg ± 5 dioptre), whilst the “bad” eye has either a minimal power lens (eg ± 0.5 dioptre lens) or the patient’s known refractive correction or c) blocked using paired cylinders of opposite signs over the good eye (eg +3 and −3) placed initially with their axes aligned to cancel each other out – then with the cylinder axis rotated by 15–20 degrees to create blurring.	However fogging is done, this is an opportunity to explain back to the patient or carer afterwards that vision in the bad eye can be temporarily improved by confusing the brain with different eye tests. “The brain thinks it is seeing out of the good eye, which is why vision improves, even though its actually seeing out of the bad eye”. It helps move the conversation away from the eye to the brain where the problem is.
**Bagolini glasses** – Where the patient perceives one light with two long striations arranged in a saltire’ cross, binocular single vision is present which should not be possible if the patient has a structural cause for visual loss.	Explanation of the mechanics of the test^a^ will be understood by very few patients – but you can nonetheless explain how you need two eyes to be working to see the cross, so like fogging its evidence that the brain’s visual pathway works better than the patient perceives it to.
**Prism dissociation testing** – through various manipulations^b^ prisms can induce diplopia in individuals with normal binocular vision. Even if the patient doesn’t experience diplopia, then the patient can be seen looking between one image and another	The test can be discussed with patients and potentially saccadic movements shown to carers as evidence that there is double vision *even though the person doesn’t experience it*, again pointing to a brain rather than an eye problem
**Stereopsis testing** – as a substitute for acuity testing. For example, ability to see a 55 degree 3-D image on the Frisby Stereotest is equivalent to 6/12 vision in both eyes	Not so helpful as a demonstration of normal vision, but discussion is another way to emphasise what, at a minimum, you might expect visual acuity to be if functional visual loss could improve.
**Visual field impairment**	
Check whether the patient actually notices a visual field problem as often they don’t. Can still be useful as ancillary evidence with a discussion	
**Tunnel vision** – eg same visual field at 1 m as at 2 m – should be conical and not a cylinder.	Explain that the brain ‘expects’ to see a tunnel which is why the patient has that response. The laws of physics mean that a field must get wider the further away it gets so the explanation is that the brain is suppressing the additional visual information to fit with its expectation. Use of a diagram may help (Figure 2).
**Spiralling and other visual fields** – including spiralling, crossing of isopters, clover leaf pattern and stacking. All common in functional visual loss but can also occur due to poor technique or retinopathy, as attention dependent.	Explain that spiralling is a feature that doctors look for when diagnosing functional visual loss. Spiralling happens because during the test the person gets more and more tunnel vision as the test goes on. Ask if they felt “spaced out” during the test and explain how the brain tends to make tunnel vision when it’s spaced out or under threat.

^a^Bagolini Glasses investigate retinal correspondence and consist of two striated lenses in a perpendicular arrangement at 45 and 135 degrees, in a lorgnette arrangement (handheld spectacles). When a light source is viewed through the lens it produces a line of light perpendicular to the striation.

^b^Prisms can be used in several ways to assess for functional vision loss in monocular cases. In severe monocular functional visual loss, a 10-dioptre vertical prism test can be used to displace an image vertically towards the apex of the prism, thus leading to recognition of diplopia [16]. Even if the patient denies experiencing diplopia, the diplopic images are difficult to ignore or ‘suppress’ and the patient can often be observed looking from one image to the other where visual acuity is near equal. Similarly, a prism reflex test, typically with a 20-dioptre horizontal prism, can be used in an adult patient to induce a motor fusion response. The prism is placed base out before one eye whilst the patient fixes on a near target. This induces diplopia and the eye behind the prism adducts towards the apex of the prism to move the prismatic image to the fovea. The fellow eye will make a corresponding abduction but due to ongoing diplopia it will make a positive fusional adduction after a second or so to restore binocular single vision. This fusional movement confirms the patient has binocular vision even if they don’t report experiencing diplopia - only patients with severe unilateral visual loss will not overcome the prism and therefore not make the second fusional movement. In unilateral cases with less severe impairment the similar monocular vertical prism dissociation test using a 4-dioptre vertical prism [17] can be used over to induce vertical diplopia whilst fixating on a high acuity target (eg single 6/6 Snellen optotype at distance). This relies on the patient reporting they observe diplopia and if so to compare the sharpness of the two letters. If they are reported as similar, then this confirms good acuity in both eyes.

**Fig. 2:**
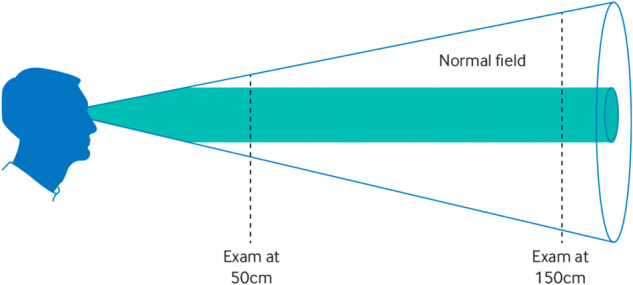
In functional visual loss a tubular visual field arises primarily because of a strong “prediction” of tunnel vision in keeping with an / predictive processing model of how the brain works. It can be explained to the patient as evidence that the brain is ‘suppressing’ vision to match an internal model. Reprinted from Stone et al. [18].

We share them here to help the reader think about the *therapeutic* opportunities that some of these tests afford, in sharing the diagnosis, explaining the mechanism of symptoms and what might be done to improve vision.

Ophthalmology affords more techniques to make a firm diagnosis of FND than for any other motor or sensory symptom. Ophthalmologists will be aware that these tests are mostly based on ‘trickery’– which the patient usually never finds out about. One important advance in the FND field more generally has been a recognition that explaining these ‘tricks’ (see Table 1) can be therapeutic. For example, Hoover’s sign of FND-related limb weakness (weakness of hip extension which returns to normal during contralateral hip flexion), or the tremor entrainment sign in functional tremor (cessation or entrainment of tremor with contralateral externally cued movements), is one of the best ways of helping people to understand and have confidence in their diagnosis. This was reflected in an article entitled ‘*Trick or Treat’* describing this process in relation to motor FND assessment [15, 16].

## Investigations – to look for comorbidity, not to make a diagnosis of exclusion

In addition to slit lamp examination, most patients will require some degree of further investigation to look for eye, retinal or neurological pathology, including optical coherence tomography, retinal imaging and autofluorescence and MRI brain and orbits with contrast. MRI is the modality of choice as CT will miss some cerebral pathologies such as demyelination. MRI is also more helpful for looking for changes compatible with conditions like posterior cortical atrophy. These tests are typically regarded as essential to ‘excluding’ other pathology which can also give the incorrect impression that the diagnosis of functional visual loss is *also* a diagnosis of exclusion.

In the presence of clear positive features of functional visual loss we recommend reframing investigations as a search for comorbidity, which we know is common in functional visual loss. However, even if something is found, if the patient has evidence of better vision than they are aware of, unless it’s cortical visual loss/blindsight, then this also means they have functional visual loss.

Try explaining to your patient, at the earliest opportunity that you have found evidence of functional visual loss, but that you need to do further investigations to make sure there is nothing else going on.

Many clinicians are reluctant to bring up the possibility of functional visual loss until every test has been completed. But by that stage, several consultations may have occurred, and a significant amount of time may have lapsed. Introducing a functional visual loss diagnosis at a late stage is harder. The patient may reasonably wonder why the clinician hadn’t mentioned it earlier or whether other things are being kept from them. Trust and transparency are an essential element in any doctor-patient relationship and should be no different for people with functional disorders.

Better management of functional visual loss involves the clinician normalising it as a condition and treating it the same way as any other neuro-ophthalmological diagnosis, which includes discussing it early as a differential when appropriate.

### Optical Coherence Tomography (OCT) scanning

OCT scanning can reveal retinal pathology in eyes which appear normal on slit lamp fundal examination. Particular attention should be paid to the outer segments of the photoreceptors, ellipsoid zone, and retinal pigment epithelium to look for subtle disruption that can be seen in occult or early retinopathy. Careful assessment of both the peripapillary retinal nerve fibre layer thickness and the macular ganglion cell layer may be especially important.

Supplementing OCT with wide-field fundus photography and autofluorescence can add further confidence that no subtle outer retinal pathology is being missed.

### Electrophysiological testing

Electrophysiological testing, which is discussed extensively in another article in this issue of Eye is often important to look for other retinal comorbidities in functional visual loss [17].

### MRI brain

We recommend an MRI brain and orbits with contrast in all patients with significant or long-lasting diagnoses of functional visual loss. It’s particularly important to consider whether your patient may have a structural cause of cortical visual loss such as stroke, multiple sclerosis, or tumour. A particular catch are patients with neurodegenerative diseases, especially posterior cortical atrophy, which can present with variable visual field deficits that may vary between assessments and not necessarily be obvious on MRI emphasising the importance of the clinical history and examination.

## Explaining functional visual loss to a patient – “do what you normally do” and “don’t be weird”

There is consensus that patient’s confidence in the diagnosis of FND and motivation to engage with rehabilitation therapy is essential for successful treatment [18]. Conversely, if a patient rejects the diagnosis, perhaps because it’s poorly explained, or because information is hard to access or understand, rehabilitation often fails. A successful explanation about FND to a patient is therefore a vital first step to allow successful treatment. In some cases, a well communicated diagnostic explanation can be, in of itself, therapeutic.

In general, what tends to go wrong in the explanations of functional visual loss, and FND more widely, is that clinicians behave and talk in a way that is at variance with their normal practice. In essence, they do things that are ‘weird’ and are perceived as such by the patient. To correct this, all that needs to happen is for the clinicians to explain this disorder in the same way as they do any other condition in neuro-ophthalmology.

Normally when a clinician explains a diagnosis, they start by naming the condition (i.e., idiopathic intracranial hypertension), give an explanation of why they think that diagnosis is correct (fulfils the diagnostic criteria), perhaps some discussion of mechanism (there is a problem with CSF overproduction) and then finally, when the time is right, a discussion of why it may have happened (metabolic disease in association with weight gain). There may be a discussion of what conditions the patient doesn’t have, especially if they had a concern about brain tumour, but those typically follow on after the identification of the actual diagnosis.

A recent US review of functional visual loss warned against “confronting” a patient with their diagnosis of functional visual loss as it is ‘rarely helpful given its aggressive and negative connotation’ [19]. Instead, management with reassurance that symptoms are likely to improve is suggested as the default option by many authors and the American Academy of Ophthalmology [19, 20]. Another recent review suggested that patients should be diagnosed with functional visual loss but should be told that their symptoms ‘do not have any anatomic or physiologic basis’ [21].

In the UK we suspect patients with functional visual loss are more frequently given a diagnosis, but even here, clinicians have traditionally often started by explaining that all the tests are normal, and that there is no reason for the visual symptoms in the eye or brain. As discussed in ‘investigations’ above, this often occurs after a long period of testing in which the patient may have an expectation that an answer is going to finally be found. There may then be an awkward moment where the patient is thinking ‘OK but what is it then?’. The clinician may then venture that these kinds of problems can occur in relation to psychological issues or stress or are ‘non-organic’. This kind of approach generally leads to a patient believing that the doctors are saying their symptoms are ‘all in their head’ or ‘imagined’, because their tests have failed to pick up a problem. When a physical symptom such as visual loss is attributed to psychological factors, most patients, as well as doctors, have ambiguity about whether that is a genuine problem, or whether its potentially feigned. The patient who has the experience of being unable to see, is understandably going to be upset and angry at the implication that they have something they might have control over, when their experience is the opposite, that they have no control over it.

What has gone wrong here is breaking of the normal rules of explanation. The clinician has not named the condition, has only explained what the patient doesn’t have, and jumped to conclusions about ‘why’ they have the problem without going through the expected stages of communication including evidence for the diagnosis and explanation of mechanism.

To correct this our primary recommendation is for clinicians to ‘do what you normally do’ when it comes to explanation of functional visual loss, the caveat being that you may not be used to giving the condition a name or explaining the nature of your testing.

### Provide a name

Diagnostic labels are signposts to information and to treatment. They can also be a burden to patients. Usually someone should only be told they have functional visual loss if they are clearly symptomatic from that or may benefit from treatment.

Be careful with the use of the term functional neurological disorder (FND). Not everyone wants or needs to have a ‘disorder’. Some people just want to know what is causing their symptoms. The name you choose should sit comfortably with you and be consistent with the information provided. Functional visual loss is, in our view, the least worst term that we currently have. It emphasises a problem with function, rather that structure of the nervous system, promoting a less dualistic framework of understanding that leaves room for multiple aetiological factors. It is however a broad term which can be confusing for patients. In contrast, psychogenic visual loss, conversion disorder and somatisation, suggest a narrow psychological aetiology for functional visual loss. This is not in keeping with broader understanding of FND which clearly does involve the brain, as well as recognising the importance that comorbid eye and brain disease play in predisposing and precipitating functional visual loss. The term non-organic should, in our view, be abandoned since human beings are wholly organic and cannot have disorders that are not based at some level on “organic” processes.

### Explain why you are making the diagnosis

In Table 1 we have outlined how nearly all the diagnostic procedures used to make a diagnosis of functional visual loss can be turned into opportunities for explanation and discussion with the patient.

The principal here is of transparency between clinician and patient, that may come as a relief to both of them. Instead of the diagnosis being handed down by the MRI scanner, OCT or electrophysiology, the process is brought back to the bedside. When successful our experience is that this is the *key* part of the diagnostic explanation. Demonstration of vision which is better when distracted or automatic compared to when the person is attending to or “voluntarily” seeing, has several immediate implications. It shows that there is a possibility of improvement, it indicates that the problem must be in the brain rather than the eye, and that changing attentional focus may play a role in treatment. It also helps the patient see that as a clinician you still believe they have a problem, even though you both now know that vision can be better than it appears to be.

In addition to the specific explanations around tests above we have provided some further ideas for ways to explain functional visual loss in general, as well as phrases that are often *not* helpful or misinterpreted in Table 2.

**Table 2:** Examples of helpful communication for functional visual loss and things to avoid– adapted from [26].

Communication issue	Example	Things to avoid
**Naming the problem**	‘You have functional visual loss – this is a common issue and I’m going to explain to you what it is’	‘You don’t have x, y, z’; ‘There’s nothing actually wrong with the eye or brain’
**General explanations of functional visual loss**	‘This is a problem related to abnormal functioning of the brain. Your eyes are sending visual signals to the brain, but the brain is not letting you experience them’	‘It’s non-organic’
**Use of metaphor**	‘It’s a bit like a software problem on a computer rather than a hardware problem’; ‘Nothings damaged – it’s just not working properly’	Comparison to everyday physical symptoms like blushing often unhelpful
**Use diagnostic tests to explain a ‘rule in’ diagnosis**	‘You have functional visual loss because I found evidence of it on the tests – let me explain.’ See all the examples in Table 1	‘All the tests are normal so that’s good, isn’t it?’
**Overcoming dualism**	Patient: ‘So, are you saying it’s psychological?’ Healthcare professional: ‘Functional visual loss is a condition that shows that the mind and the brain are one and the same thing.’	‘It’s a psychological problem’. Psychological factors are risk factors rather than causes
**Talking about mechanism before aetiology and introducing hope**.	‘The tests we have discussed show that you have the potential for experiencing better vision, but we need to find a way of retraining your brain so that this can happen’	Not talking about mechanism!
**Discussing physical and mental health risk factors**	‘The fact that you have diabetic retinopathy, have bad migraine that affects your vision and the mental health problems you described are all likely to be relevant to why this has happened’	‘We need to figure out what your psychological trauma has been so we can treat you’
**Talking about photophobia**	‘People with functional visual loss often have light sensitivity. We think it’s your brain trying to shut down visual input when its feeling overwhelmed’	‘You must take those dark glasses off – they are just making the problem worse’
**Providing information**	Copy your letter to the patient. Signpost to resources on functional visual loss and FND – eg neurosymptoms.org, fndhope.org	
**Prognosis**	‘This is not an easy problem to put right, but it does have the potential to improve, and many people do make a good recovery.’	‘Because there’s nothing wrong then it should just get better’
**Orthoptic treatment**	‘My orthoptic colleague can spend longer explaining things and going through the tests with you. They may be able to start using the tests to help glimpse better vision’	
**Psychological referral**	‘Psychology/Psychiatry can sometimes help people with functional visual symptoms to look for risk factors such as anxiety or depression that could be making the brain problems worse. What do you think?’	‘This is a mental health problem; I’m sending you to psychiatry’
**Talking about hypnotherapy**	‘Hypnotherapy has a strong scientific basis. It can be a way of putting the brain in an altered state that might allow you to have better vision. You can be taught self-hypnosis that might help’	‘You could explore complementary therapies like hypnosis’

The use of metaphor may be helpful. The brain is not a computer and ultimately every change in function must have some change in structure, but analogies to software malfunctioning (or a piano or car out of tune for those that don’t understand software) may help. For some people we have found it helpful to refer to the 2015 Pixar film ‘*Inside Out’* in which there are different characters in charge of different brain functions. In functional visual loss it is as if the character in control of vision has decided to go on strike.

Instead of jumping to a discussion of aetiological risk factors, we recommend starting with a discussion about the mechanism of symptoms first, for example a clinician may say “Your eyes are sending visual signals to the brain, but the brain is not letting you experience them” – see Table 2. This is what ophthalmologists and neurologists are used to doing. For example, you wouldn’t try to explain to a patient *why* they had developed multiple sclerosis or Parkinson’s disease without first explaining that these are conditions associated with inflammation of the brain or loss of dopamine.

Making referrals to other specialists should also be done with care. If the basic problem is the brain not working, then it’s important to consider a wide range of techniques that might help the brain work better again. This may include psychological assessment and therapy, based on the model of what we think causes functional visual loss or other treatments like hypnotherapy that may need some careful explanation to avoid misunderstanding about what’s being suggested.

A concern many ophthalmologists may have in discussing functional visual loss is that any consultation may be consumed by discussing psychological factors or adverse life events which they may feel they lack the skills or time for. As outlined in Fig. 1 adverse life events or mental health diagnoses can be seen as risk factors and not the cause of the visual loss. Given limitations on time and the distress that comes with discussing these topics there is often no need to discuss these aspects in detail especially in the first diagnostic consultation and especially if the patient is not keen to do so. The priority in initial consultations is to reach a diagnosis based on clinical evidence and explore mechanisms of symptoms with patients. Integrating that with more complex risk factors where present takes time, should occur at the patient’s own pace and is often a goal of treatment, rather than something that can be ‘solved’ at the first appointment.

Providing written materials and onward signposting can be helpful for people. Copying your letter to the patient is another way of promoting transparency and educating the patient. At present, there aren’t detailed materials for people with functional visual loss, but they could be signposted to FND resources such as neurosymptoms.org (made by JS one of the authors), or patient-led organisations such as FND Hope, FND Action. The MyFND app can also be appreciated by patients. Be careful however, not to simply recommend a whole FND resource if the patient has only visual symptoms. It may be frightening for them to read about seizures or paralysis. Instead, direct them to the specific resources about functional visual symptoms. There is also now an international FND Society for health professionals, all of which lends legitimacy to the condition (fndsociety.org).

### Following up the patient and triaging for treatment

Many patients report bewilderment at being given a complex diagnosis such as FND and then being immediately discharged. Again, is this what you would normally do for someone with visual loss due to a structural condition? A follow-up visit allows the neuro-ophthalmologist to see if the patient agrees with the diagnosis given and whether now is the correct time for treatment. Is the patient actually troubled by their visual loss? Do they want further treatment at this time?

If the diagnosis has been raised at the first visit, then there may be investigations to review anyway at the time of a follow up. If treatment is being attempted then experience more broadly with FND suggests that therapists notice greatly enhanced treatment responses if the diagnosing clinician or a health professional who can explain the method of diagnosis, remains involved [22].

## Further Management

The lack of research into the management of functional visual loss is striking. A study of 10 patients used transcranial magnetic stimulation with improvement in 9 patients [23]. A case series of 33 paediatric patients reported that 28 recovered with suggestion therapy [24]. The Edinburgh FND research group published a detailed description of the more multi-faceted approach, describing visual recovery in two individuals with complete functional visual loss, with a focus on explanatory models, combined with transcranial magnetic stimulation and hypnotherapy [25]. This does not provide an evidence base on which to recommend treatment so what we have written here is based on experience.

While we advocate for ophthalmologists to stay involved, treatment of FND including functional visual loss should be a multidisciplinary endeavour.

**Fig. 3:**
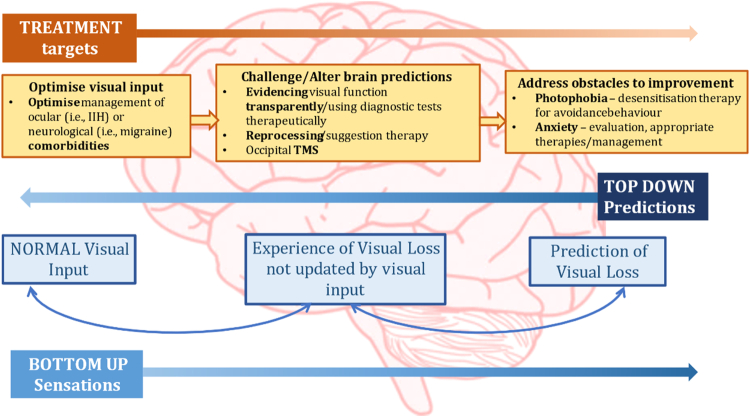
A model of functional visual loss highlighting the mismatch between “top-down predications” and “bottom-up” visual input and how potential therapies can target this.

The predictive processing model of functional visual loss described earlier opens the possibility for newer treatments for functional visual loss (Fig. 3), some of which may be enhanced by education and techniques at the bedside.

### Encouraging the patient and carers to look out for evidence of briefly better vision

Instead of ‘glossing over’ the variability of vision seen in functional visual loss, we have found it helpful to ask patients and carers to look out for instances of better vision. In most cases these have already been noticed, especially by carers. For example, a recent patient of ours went into a shop and saw a t-shirt of the cartoon character ‘Bluey’ that she thought would look lovely on their baby. Instead of feeling bewildered or worried that people might think she was making up her symptoms, she and her partner had been encouraged to see this as a sign of visual pathways working better at a moment when she was thinking about someone external, and something to reflect and feel positive about.

### Orthoptic therapy using diagnostic tests as treatment

Our early experience using diagnostic tests to help patients and their carers glimpse normal vision has been positive. Based on other forms of FND it seems likely that this can form the basis of an initial approach to therapy that can be started by neuro-ophthalmologists but delivered most effectively by orthoptists. Essentially the overweighted strong ‘prediction’ of visual loss which is not challenged by normal visual input can be challenged by the kind of visual input created during testing. Orthoptists bring a combination of knowledge of the visual system, understanding of the mechanisms of testing but also the ability to carry out a longer assessment and begin to integrate other elements of treatment.

**Fig. 4:**
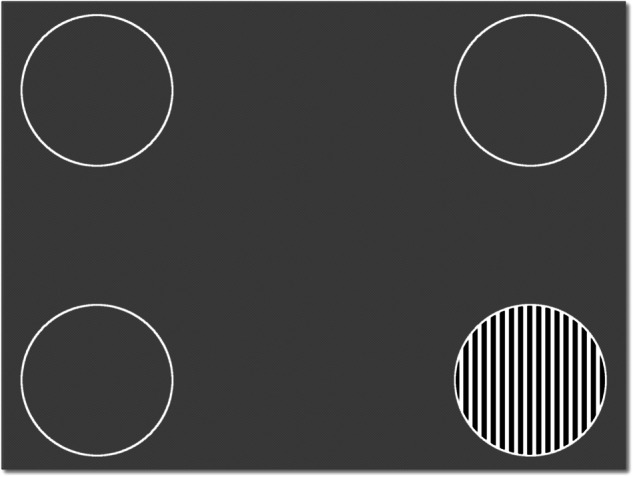
The ‘Peekaboo’ app is an example of how technology for visual testing could be used to help treatment of visual loss. Observing saccades towards the striated target enables examiner and carer to infer the patient has seen it, even if the patient reports they do not see it. In addition, the patient can be asked to guess where the target is by touching the screen even when they can’t see it. This can help gauge/ prove to the patient whether their ‘brain’ can still see, even if they don’t have visual experience.
- Explaining to patients that seeing a “saltire” cross in Bagolini glasses indicates that both eyes are receiving vision even if one eye is blocked in normal circumstances.
- Following tests of stereoscopic function such as the Frisby stereo test, explore whether patients would wish to practice with 3-D picture books, stereograms or even 3-D films to build confidence that both eyes are functioning.

Some examples of this may include:

- Swapping between phone font vision and test vision to see if better test vision can be achieved.
- Testing visual acuity using single letter optotype and comparing to acuity when letters are together.
- Repeat the fogging test, but therapeutically until the patient is seeing out of their “bad” eye and then provide positive feedback or carry out visual tasks.
- Using an app like ‘peekaboo vision’ (see Fig. 4) to see if the patient can become aware of their own visual choices. Ask the patient to guess where to tap the screen, even if they don’t perceive it and provide feedback about accuracy. Some individuals may show below chance performance which is also an opportunity to discuss that the brain must be seeing something in order to choose the opposite response.

There is a large potential here for innovative therapies that use these principles, in a similar way to what has successfully been described for physiotherapy in functional motor disorders [26, 27]. They could become automated using gaze tracking or mobile phone technology.

### Managing eye and brain co-morbidity

Optimising these comorbidities can have the effect of improving functional visual comorbidity by improving ‘bottom up’ visual input and also improving confidence and optimism in vision.

Migraine is commonly co-morbid with FND and can often improve if migraine is better treated. Although ophthalmologists are not headache specialists, it’s important to recognise that migraine is often undertreated. For example, is the patient taking appropriate acute migraine treatment? (i.e., a combination of high dose NSAID, anti-emetic and a triptan). Is the patient overusing analgesia? (i.e., use of painkiller >10 days per month, with particular focus on overuse of codeine). If they are having frequent headache days, is the patient on an appropriate preventative for migraine at the correct dose? Botulinum toxin injections, CGRP monoclonal antibodies and CGRP receptor antagonists or “gepants” have greatly expanded potential therapies for migraine [28].

Other types of functional neurological disorder (for example, functional movement disorder or seizures) and functional disorders (for example irritable bowel syndrome or fibromyalgia) are more common in patients with functional visual loss [29]. An integration of these conditions as having overlapping mechanism is usually helpful in management [6].

### Management of photophobia

Photophobia is common in functional visual loss. A study looking at 34 consecutive patients who wear sunglasses in clinic found that 79% had functional visual loss, with nearly all of them citing photophobia and/or headache [30]. The authors of the paper suggested this might be a phenomenon designed to emphasise visual loss or copy famous individuals with blindness such as Stevie Wonder. These authors considered use of sunglasses was not ‘reasonable’ in this group. However, in our experience, the behaviour develops primarily because of photophobia which can become extreme, for example sitting in a darkened room because taking the glasses off is uncomfortable. The use of sunglasses is therefore reasonable to the patient and has short term benefits, although in the longer term makes things worse. We recommend starting by explaining that the brain is sensitive, but the more you wear sunglasses the more sensitive it becomes. Management involves graded sensitisation to light which may involve a structured plan to have reductions in opacity of sunglasses or spend longer and longer without sunglasses. This kind of graded exposure is generically familiar territory for clinical psychology, for example in relation to insect or flying phobia, but they may need support to understand that this approach can also work for photophobia. A clinical psychologist may be helpful but is not always essential to help people wean themselves off sunglasses in our experience.

### Psychological assessment and therapy

More detailed psychiatric assessment, typically from a liaison or neuropsychiatrist familiar with FND, can be valuable in assessing for risk factors and comorbidities. Treatment of common psychiatric comorbidity such as anxiety, depression, PTSD or obsessive-compulsive disorder with psychological therapy or medication can sometimes help to improve or resolve FND symptoms. Assessment for developmental differences such as autism, ADHD or emotionally unstable personality traits may be an important part of reaching a formulation. Psychologists can also assist with graded exposure for those wearing sunglasses who have photophobia (as above) and integrate psychological experiences and symptoms into their experience of functional visual symptoms. Individuals with trauma may benefit from a trauma focused approach to therapy that helps explain their brains tendency to dissociate in response to threat and find different ways to respond to medically or psychologically threatening sensations. Approach to psychological treatment here can borrow from what we have learnt about the approach to other FND symptoms, such as seizures and limb weakness [31].

Our experience with patients who do recover vision is that it helps to prepare them for how they are going to explain a recovery to others. Some patients worry that if they recover people will think they were “making up their symptoms all along”. Most people are aware that visual impairment is hard to treat unless you have an operation or medical treatment. We advise our patients to tell friends and family that a truthful statement they are undergoing therapy which aims to “retrain the brain” and improve vision is helpful in advance.

### Hypnotherapy, suggestion and relaxation during education

Suggestion and relaxation techniques are components of many therapies including mindfulness, eye movement desensitization and reprocessing (EMDR) therapy and hypnotherapy. They may all enhance the process of ‘altering brain predictions’ about vision, perhaps during a visual task such as eye tracking.

Hypnotherapy and suggestion have been tested in over 30 studies in the FND literature including 5 randomised controlled trials [32]. In most of these studies patients showed benefit. Some of these studies had mixed patients with functional visual loss [33] or where single case reports so are hard to draw conclusions from. One study in particular focused on a suggestion technique in 8 consecutive patients with functional visual loss (2 of whom were over 16). The patients were given a lengthy but non-specific visual task to carry out at home along with a strong suggestion of improvement [34], with most reporting improvement or having evidence of improvement on testing. It’s likely that the outcome in a younger group like this would have been better anyway.

Our own experience is that hypnotherapy, in the right hands, can be helpful but it is essential that the practitioner understand the principles of diagnosis of functional visual loss to integrate this into their approach.

### Other novel treatments

#### Transcranial magnetic stimulation (TMS)

TMS has been trialled by researchers treating motor FND. It can undoubtedly be helpful, although it appears that cranial TMS is just as effective as spinal TMS suggesting the effect is more about demonstrating movement in a limb that is paralysed rather than neuromodulation [35]. Occipital TMS generates a visual experience of phosphenes. Our experience is that it can be a useful adjunct to support treatment, especially for bilateral visual loss [25]. The phosphenes provide evidence that the visual cortex can produce visual experience when stimulated. It also likely has a strong suggestive value but even that can be discussed transparently.

#### Therapeutic sedation

Using sedation and anaesthesia has a history of treatment in FND going back over a century. One of the authors (JS) described a case series of patients with functional paralysis, dystonia, coma and other symptoms with resolution of symptoms in some [36]. We have not used this for functional blindness, but it is a technique that would be reasonable if others failed and if the patient had a good relationship with the treating clinician.

## Other management issues

### Managing paediatric patients

The literature consistently demonstrates better outcomes for children and young people compared to adults with FND. In younger children, especially if the problem is recent onset, an explicit diagnosis may not be so necessary and reassurance that things are likely to improve may be reasonable depending on the family and school situation. In teenagers the management is usually quite similar to adults. Liaison with school at an early stage often helps with management.

### Register visually impaired or not?

In the UK survey of 119 ophthalmologists mentioned earlier, 22% considered that those with functional visual loss should not be registered as visually impaired. This is not a black and white issue and is a similar question to whether people with severe disability from motor forms of FND ought to be provided with wheelchairs and disability aids. A consensus view occupational therapy guideline concluded that for individuals with persistent disability (who were not in active treatment) that quality of life should be maximised and likely benefit and gain greater independence with disability aids such as a wheelchair [37]. Similarly, if someone has persistent visual loss then it is likely to be helpful to be registered blind or partially sighted. However, if the history is quite short and they are engaged in treatment, it may be counterproductive to be encouraged to rely on visually impaired aids or to be registered blind.

### Unusual functional visual agnosias

We have seen a number of unusual visual symptoms in people with FND which have not been well documented in the literature. This includes a 14-year-old who couldn’t see letters or numbers but could see symbols. A 26-year-old whose vision became black and white at the same time as she became severely depersonalised, and a 15 year old who developed a problem reading individual lines of text or graphs but could do so if they were isolated from other text. These agnosias, and possibly functional visual apraxia presentations deserve further study. There is overlap between these acquired visual symptoms and developmental visual symptoms.

## Conclusion

Functional visual loss is a relatively common clinical issue for a neuro-ophthalmologist. The diagnosis is usually relatively straightforward for an experienced clinician, but the management has not been well described. With greater experience of wider functional neurological disorder subtypes, the time is now ripe to apply similar principals to patients with functional visual loss. A conventional approach to labelling, explaining the method of diagnosis and mechanism of symptoms to people with functional visual loss works much better than one which emphasises the normality of tests or psychological risk factors. There are promising avenues to develop new types of treatments and multidisciplinary therapy for functional visual loss.
